# Institutional Questions

**Published:** 1900-02-17

**Authors:** 


### INSTITUTIONAL QUESTIONS.

In an answer to a question last week the price of "Cottage Hospitals," by Sir Henry Burdett (Scientific Press), was erroneously given as 5s. It should have been 10s. 6d.

### Compensation for Accident to Hospital Employees.

A Correspondent asks: Could you inform me whether committees of hospitals are legally bound to compensate their employees in the event of injury or death resulting from accident when in their employ; and, if so, whether such liability would extend to all employees, or only to certain of them.

*** The point raised seems to us to be a legal one, in regard to which the advice of a solicitor should be taken. Much depends upon the nature of the work. The Workmen's Compensation Act only applies to certain kinds of employment, especially kinds in which machinery is employed. So far as we can see, the only circumstances under which hospital authorities would be liable under this Act would be the very rare cases in which they undertook on their own behalf, that is, without a contractor, the repair or demolition of buildings by aid of machinery, or perhaps in steam laundry work. Still it must be remembered that the passing of this Act did not in any way affect the right of every employed person to compensation for accident resulting from the negligence of the employer; and there can hardly be a doubt that the committee of a hospital stands exactly in the same position as any private person in this respect. Clearly it is a question for a lawyer.

